# Aortic intramural hematoma and classic aortic dissection: two sides of the same coin within the acute aortic syndrome for an interventional radiologist

**DOI:** 10.1259/bjrcr.20210019

**Published:** 2022-03-09

**Authors:** Pietro Pitrone, Antonino Cattafi, Giampiero Mastroeni, Francesco Patanè, Fabrizio Ceresa, Giuseppe Nirta, Italo Giuseppe Bellone, Enrico Monsù, Maria Ludovica Carerj, Alessandra Coglitore

**Affiliations:** 1 Department of Biomedical and Dental Sciences and Morpho-functional Imaging, University of Messina, Messina, Italy; 2 Radiodiagnostic Unit, A.O. Papardo, Messina, Italy; 3 Department of Cardiac Surgery, Azienda Ospedaliera Papardo, Messina, Italy

## Abstract

Management of acute type B aortic intramural haematoma (AIH) still represents a challenging issue. Although most resolve spontaneously or with conservative therapy, several cases of AIH may complicate into classic aortic dissection with subsequent risk of aortic rupture and visceral malperfusion, thus needing urgent or preemptive thoracic endovascular aneurysm repair (TEVAR). Despite the long-term aorta-related survival, TEVAR might lead to graft obstruction, migration, infection, stroke/paraplegia, visceral ischemia, endoleak and, last but not least, retrograde aortic dissection (AD), frequent in the acute phase and associated with a high mortality risk. In order to highlight such a close relationship between AIH and AD and the possibility to perform endovascular treatment, we report the experience of an adult female patient with an aortic intramural haematoma evolving into a classic aortic dissection. Despite successful thoracic endovascular aneurysm repair (TEVAR), our patient developed an aortic dissection type A at one month with subsequent indication for cardiac surgery still representing the elective approach in case of pathologies including the ascending aorta. Thus, the aim of our discussion is to create a debate on the most appropriate management for the treatment of descending AIH.

## Summary

Aortic intramural haematomas (AIH) typically occur in older females with cardiovascular risk factors and usually involve the descending aorta.^1^ Although traditionally considered the result of a haemorrhage of the vasa vasorum, AIH is now defined as a thrombosed aortic dissection.^2^ Presenting symptoms such as chest or back pain are non-specific; so is elevated plasma D-dimer which, however, may also imply extension of a pre-existing AIH or dissection. Computed tomography (CT) remains the gold-standard technique for diagnosis, follow-up and detection of complications. In addition, CT provides important prognostic information when measuring parameters like the maximum haematoma thickness (MHT) and the maximum aortic diameter (MAD)^1^ or evaluating “ancillary findings”.^3^ Drawbacks are high radiation-doses, contrast-induced nephrotoxicity and artefacts from motion or beam hardening. Thus, several MRI techniques were developed, from the traditional time of fight (TOF) and phase contrast (PC) to the newest cardiac-gated 3D fast-spin-echo, arterial spin labelling and balanced steady-state free-precession; new contrast-enhanced techniques include time resolved imaging of contrast kinetics (TRICKS) MR angiography (MRA).^4^ However, since mural thrombi and calcifications are not well visible, MRI still represents a second-choice diagnostic tool.^5^ In the absence of univocal guidelines and due to the self-limiting nature of the disease, patients with stable haemodynamics and no complications may benefit from medical therapy only^6,7^; nevertheless, AIH may also complicate into aortic dissection or rupture, both associated with visceral malperfusion (*e.g.* stroke or myocardial infarction).^8,9^ Thoracic endovascular aneurysm repair (TEVAR) may be performed with either curative or preventive purposes. In the first case, signs of impending AD like acute or significant intimal erosions or considerable aortic enlargement are considered^1^; the second choice comes from the observation of a lowered aorta-related mortality.^7^ The rationale of TEVAR is to exclude and favour the thrombosis of the false lumen; it seems that the earliest it is performed, the higher the likelihood of fully re-expansion of the true lumen.^3^ Complications include graft obstruction, migration, infection, stroke/paraplegia, visceral ischaemia, endoleak or retrograde aortic dissection. The latter, consisting in injury of the innermost layers of the aorta with subsequent blood flow between intima and media, is frequent in the immediate postoperative period and associated with a high mortality risk.^10^ For this reason, when dealing with management of AIH type B, a careful risk-benefit assessment must be done.

## Clinical presentation

**Figure 1.**
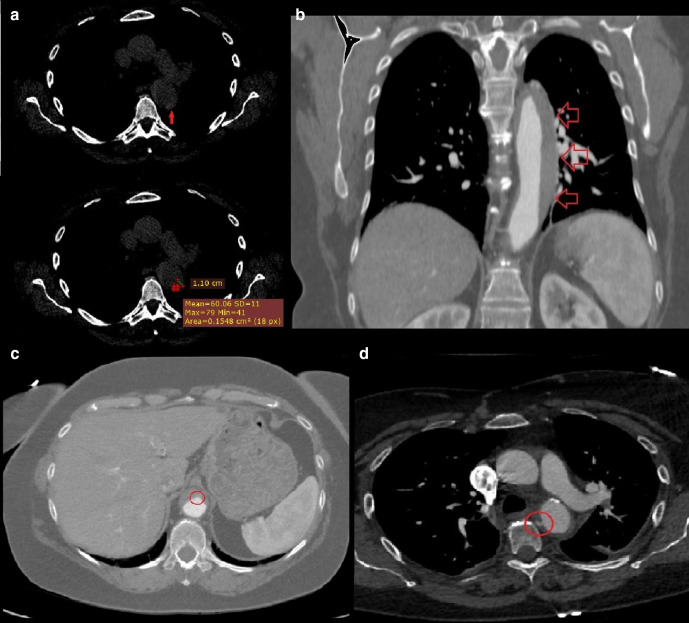
(day 1): (a) Non-enhanced CT axial scan at the level of the origin of the left pulmonary artery: a 11-mm crescent-shaped hyper attenuating (60 HU) thickening within the wall of the descending aorta on its lateral aspect is seen. (day 4, b) Contrast-enhanced CT coronal scan at the level of thoracic aorta: we appreciate the approximate extension of the above-described aortic thickening, that is, from the left subclavian artery to the celiac trunk. (c) Contrast-enhanced axial CT scan at the level of the upper abdomen: a small bulge of contrast is seen on the anterior aspect of the abdominal aorta, likely representing the “exit tear” of an aortic dissection whose thrombosis has led to the development of the AIH. (day 4, d) Contrast-enhanced CT axial scan at the level of the origin of the left pulmonary artery: a focal blush within a thrombotic lesion (7 × 3 mm, with a millimetric “peduncle”) on the medial aspect of the descending aorta is seen, likely representing the “entry tear” of the above-mentioned aortic dissection.

A 58-year-old female with mild precordial pain is transferred to the Department of Cardiac Surgery of Our hospital (day 1) with a diagnosis of AIH type B, well visible on unenhanced CT scans (Figure 1a). Clinical conditions and haemodynamics are stable with minimal infusion of nitroglycerin.

## Differential diagnosis

Pathologies of the aorta with similar clinical, laboratory and imaging presentations include classic aortic dissection, mural thrombus, aortitis and incomplete dissection.

## Investigations

**Figure 2.**
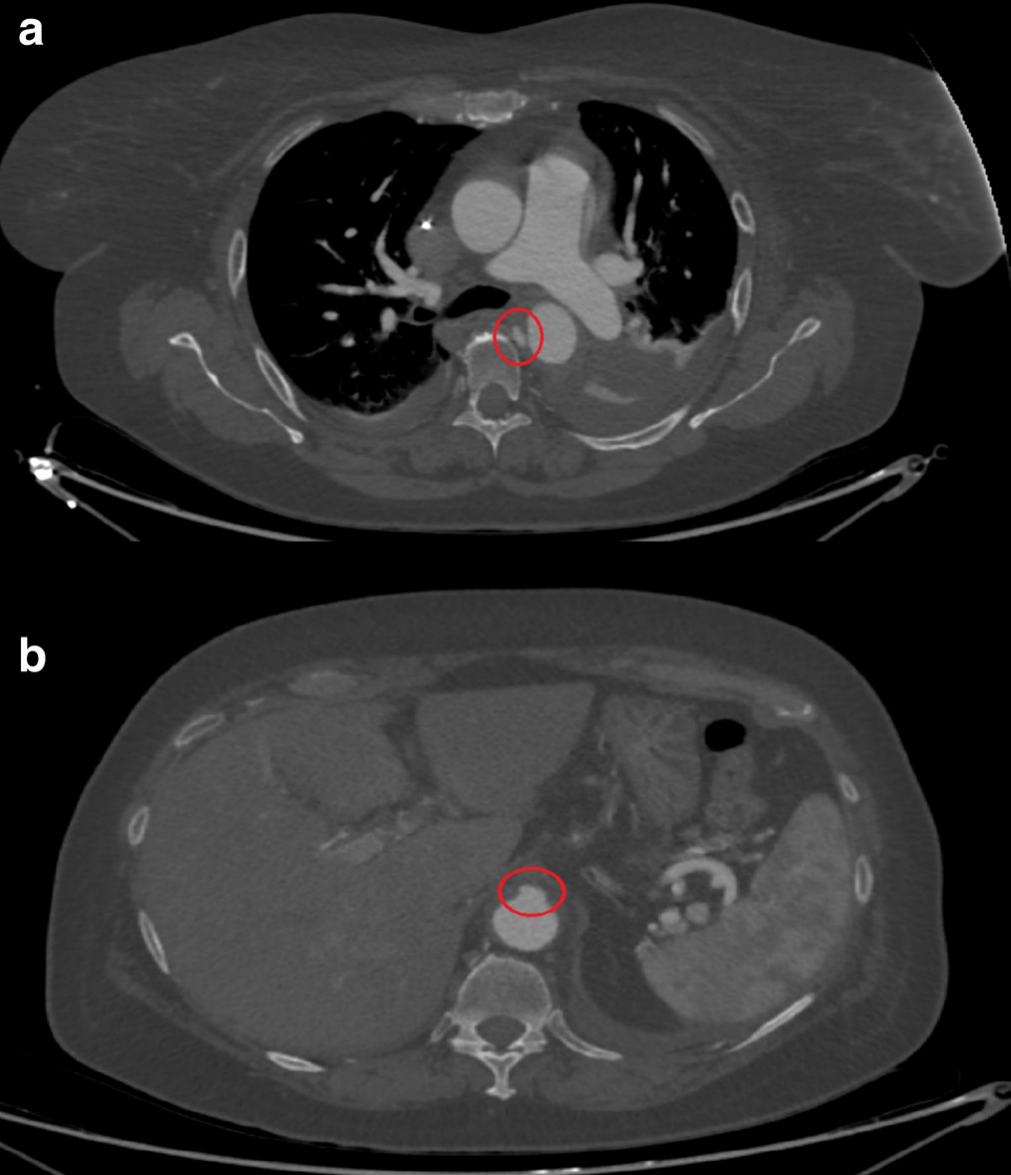
(day 8): Contrast-enhanced CT axial images at the level of the bifurcation the pulmonary trunk (a) and the upper abdomen (b): enlargement of both the entry (a) and the exit tears (b) is seen, indicating impending aortic dissection or rupture.

The following contrast-enhanced CT (day 4) clearly shows the real extension of the AIH, that is, from the left subclavian artery to the celiac trunk (Figure 1b). In its most distal aspect, above the origin of the celiac trunk, a small bulge of contrast is seen, likely representing the “exit tear” of a thrombosed aortic dissection (Figure 1c). Cranially, on the medial aspect of the descending aorta, a focal blush within a thrombotic lesion is seen, likely representing the “entry tear” (Figure 1d). TEVAR is scheduled and further CT is performed (day 8), documenting enlargement of both the entry and the exit tears (Figure 2a and b).

## Treatment

**Figure 3.**
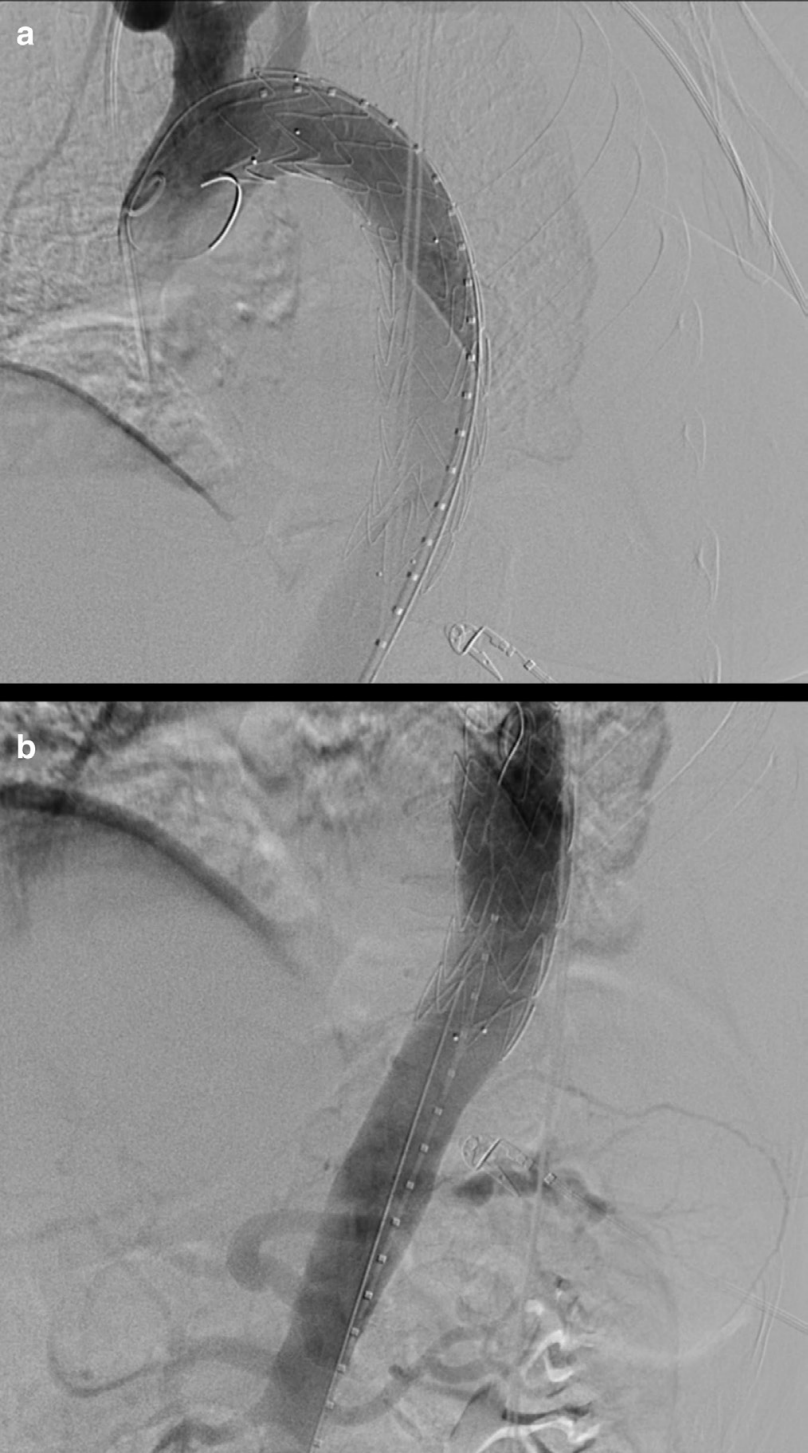
(day 8): Digital subtraction angiography images at the level of the cranial (**a**) and caudal (b) segments of the thoracic aorta (a): optimal positioning of a Valiant Navion 34 × 174 mm stent graft (Medtronic Vascular, Santa Rosa, Calif) is documented, with the “free-flow” portion at the origin of the left subclavian artery (with caudal aortic diameter measuring 29 mm).

Patient is transferred to the hybrid angiographic room on the same day and a Valiant Navion 34 × 174 mm stent graft (Medtronic Vascular, Santa Rosa, Calif) is deployed, with the “*free-flow*” portion at the origin of the left subclavian artery (Figure 3).

## Outcome, follow-up and discussion

**Figure 4.**
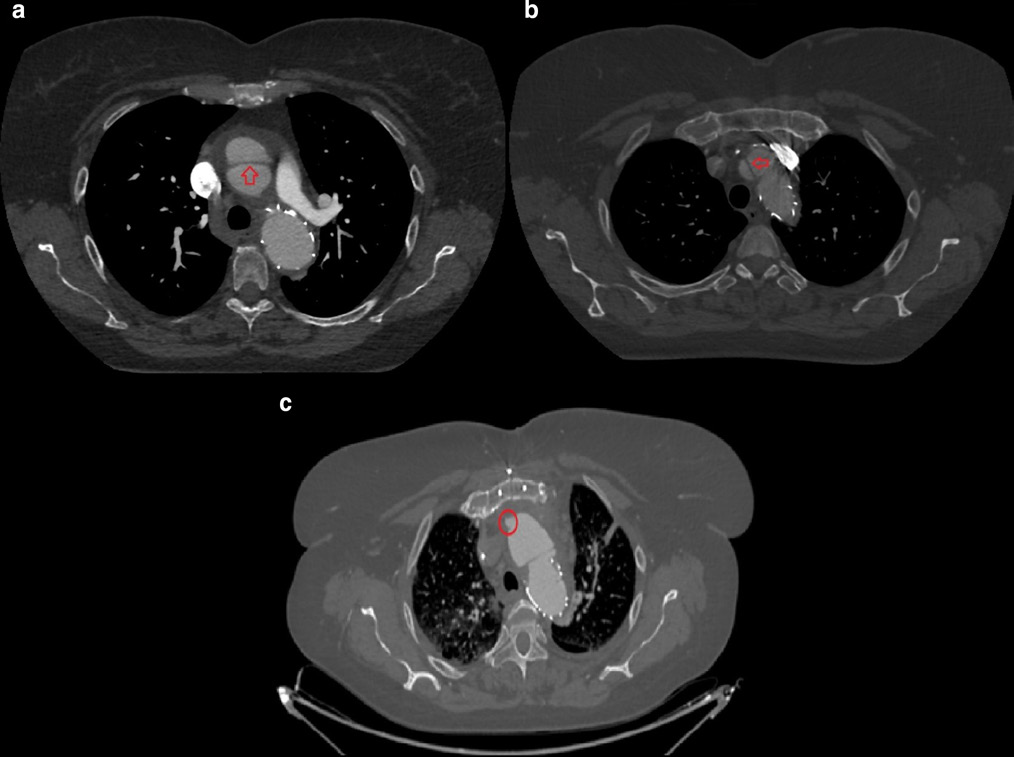
(day 42, **a, b**) Contrast-enhanced CT axial scans at the level of the origin of the left pulmonary artery: an intimal-medial flap cranially to the stent graft extending to the origin of the right coronary artery is seen and is consistent with “retrograde aortic dissection type A”. (day 52, (c) Contrast-enhanced CT axial scan at the level of the aortic arch following cardiac surgery: a small bulge of contrast on the antero medial aspect of the ascending aorta is seen, likely representing the “entry tear” of above-mentioned retrograde dissection type A.

A one-month follow-up is scheduled: the following CT (day 42) shows an “aortic dissection type A” cranial to the stent graft, extending to the origin of the right coronary artery (Figure 4a and b). Open surgery is thus performed, with substitution of the ascending aorta with a 28-mm dacron tube graft (Maquet Hemashield, Baden-Württemberg, Germany) and of the arch-descending aorta with a Thoraflex 30/34 Hybrid Plexus prosthesis (Vascutek, Renfrewshire, Scotland, UK). The next control CT (day 52) shows no complications; a cranial bulge of contrast, which might have represented the entry tear of the dissection type A, is visible (Figure 4c).

Aortic intramural haematoma represents the result of a thrombosed aortic dissection.^1^ A haemorrhage of the vasa vasorum was traditionally advocated, and many authors have recently proposed a role in the genesis of those AIH with no visible “tears”.^2^ It shows a marked predilection for old females with cardiovascular risk factors and mostly affects the descending aorta (Stanford type B). Abrupt and intense “aortic pain” at the chest or at the back (associated with type “A” and “B”, respectively) is typical; the elevation of D-dimer suggests new-onset AIH but also extension or evolution into dissection. Computer tomography, representing the gold-standard technique, shows circular or crescent hyperattenuating (60–70 HU) non-enhancing thickening (usually over 7 mm) within the aortic wall and displacement of intimal calcium. It also has an important prognostic role when measuring MHT, MAD and ulcer-like projections (ULPs), predicting a worse prognosis when over 11 mm, 50 mm (in width) and 10 mm (in depth), respectively.^1^ Ancillary findings like pleural or pericardial effusions, periaortic and mediastinal fat stranding within the first 24 h seem associated with poor outcomes. Finally, CT is employed for follow-up and potential complications.^3^ However, high radiation-doses, risk of contrast-induced nephrotoxicity, motion and beam hardening artefacts still represent an issue.^4^ Although the use of paramagnetic contrast material shortening the T1 relaxation time improves contrast between blood vessels and background, mural thrombi or/and calcifications are not well visible on MRI.^5^ Conventional techniques include TOF and PC, needing long acquisition times and with limited accuracy for small vessels; newer non-contrast techniques include cardiac-gated 3D fast-spin-echo, arterial spin labeling and balanced steady-state free-precession, showing better image quality and shorter examination time but associated with flow artefacts. An example of contrast-enhanced technique is TRICKS MRA, a modified 3D gradient echo sequence; multiple 3D volumes are acquired during the passage of contrast agent bolus: a DSA-like dynamic filling of the arteries and veins is obtained, with an oversampling of the centre of k-space and an under sampling of the periphery. Its high temporal and spatial resolution allows highlighting of the arteries alone eliminating venous contamination; motion artefacts are lower than with non-contrast MRA and a larger field of view is covered. In addition, a small contrast dose is required since acquisition starts at the same time of bolus injection.^4^ Many aortic diseases may mimic AIH: classic AD has patent flow in the false lumen and mural thrombus is not hyper-attenuating. Aortitis causes a complete circular parietal thickening, does not show ULPs or intramural blood pools and determines strong PET-CT positivity. Finally, an incomplete dissection does not cause separation of the medial layers.^1^ Although most of AIH (70%) resolve spontaneously, a 4–10% mortality and a 57% complication rate are reported.^8,9^ To date, there are no univocal guidelines about the treatment of AIH type B. Patients with stable haemodynamics and no complications should be managed with beta-blockers and nitroprusside, keeping systolic pressure under 120 mm Hg.^6,7^ On the contrary, acute ULPs, intimal erosions over 20 mm in width or 10 mm in depth, refractory hypertension, uncontrolled pain, progressive pleural effusion, MAD over 45 mm all represent indications for a surgical/endovascular treatment.^1^ TEVAR prevents aortic rupture and visceral malperfusion and decreases significantly aorta-specific mortality at 5 years^7^; the aim is to occlude the entry tear (favouring thrombosis within the false lumen) and to re-expand the true aortic lumen.^1^ After CT-angiographic evaluation of femoral-iliac systems and intercostal arteries (whose extensive coverage may lead to spinal cord ischaemia and postoperative paraplegia, both related to the artery of Adamkiewicz), an appropriate stent-graft is chosen. A 20-mm-long proximal and distal seal zone are mandatory to avoid endoleak or device migration and a variable oversize must be applied according to each manufacturer’s recommendations; potential coverage of one or more of the arch/abdominal aorta branches may be avoided using the newest fenestrated grafts. Gore (Flagstaff, AZ) TAG devices are made of polytetrafluoroethylene with a nitinol exoskeleton and are suitable for small diameter, tortuous and tapered aortic anatomies. Cook (Bloomington, IN) Zenith TX2 devices are made of Dacron with a stainless steel z-stent exoskeleton and present steel barbs on their proximal component (for a better fixation and lower risks of “bird-beak” effect) and bare metal stent distally. Medtronic’s (Oakbrook, IL) Valiant devices are polyester grafts with a nitinol exoskeleton, thus showing high flexibility. The Bolton (Sunrise, FL) Relay consists of a polyester/nitinol graft with a support-longitudinal nitinol bar available in straight/tapered configuration. Contraindications to TEVAR mainly include inadequate proximal/distal seal zones, aortic tortuosity, difficult vascular access, extremes of aortic diameter or non-sterile field. The procedure is performed under general or spinal anaesthesia, since femoral access through large sheaths (20 to 26 Fr) requires percutaneous groin cut down; admission to the ICU for up to three days post-operatively is advised for haemodynamic and neurologic monitoring. The patient is discharged home within 1 week and graft surveillance (positioning and possible endoleak) with CT is performed at 1 month, 6 months and then annually. Technical success is around 98%, with need for secondary interventions in 3.6–4.4% and a 1.9–2.1% perioperative mortality (versus 5.7–11.7% of open repair)^7^; prompt endovascular repair prevents aortic wall remodelling and is thus associated with a higher success rate.^3^ The main complications include obstruction, migration (1 to 2.8%, associated with aortic tortuosity and graft oversizing), infection, endoleak (3.9 to 15%) and aortic dissection; the last one, in particular, seems secondary to the chronic radial force associated with the device (especially if oversized) and the subsequent thinning of the media.^10^ Other major complications are stroke (4–8%, due to embolisation to the carotid artery, coverage of the left subclavian artery or embolisation via the subclavian), paraplegia (3–5.6%, due to spinal cord ischaemia) and visceral ischaemia (if the celiac artery origin is covered). Access complications like iliac rupture are managed by balloon occlusion and eventually open surgery. Post-implantation syndrome with fever, leukocytosis and pleural effusions in the immediate postoperative period is associated with endothelial reaction.^7^ Back to our case, despite the discrete clinical conditions and the low burden of the disease (low MHT, MAD and erosions), the appearance of a significant “entrance tear” (10 mm) and the witnessed “instability” of our patient’s tomographic picture over time suggested a prompt TEVAR, with immediate success but with development of a proximal AD at one month. Given such complications, did we act properly? What other approach could we have chosen? Should we have kept medical therapy only and monitored the patient, waiting for a spontaneous regression (an intramural blood pool/IPB often indicates an incomplete and temporary reabsorption of an AIH) or/and planning a more appropriate elective treatment^11^? Was the graft (34 mm large, with the diameter of the aorta just distal to the left subclavian artery measuring 29 mm) slightly bigger than necessary? Should we have demanded the patient for first-line cardiac surgery, keeping in mind that AAS always reflects a pathology of the whole aorta? A review by Tanaka et al^12^ about management strategies in acute type B aortic intramural haematoma states how, despite the better prognosis than AD, rapid growth and signs of impending rupture in two successive imaging studies represent a valid indication for treatment. However, a 7.4% incidence of retrograde type A dissection after TEVAR for type B IMH is reported, especially in the acute phase, with a 34% mortality risk. For this reason, preemptive TEVAR in uncomplicated AIH on the sole basis of improved aorta-specific survival and delayed disease progression should be avoided.

Whatever the choice of management, a narrow CT follow-up is mandatory, since serious complications like AD might occur even when few suggestive symptoms are present, as demonstrated by our experience. An AD transforming into an IMH whose treatment causes a second AD: not even one association within the AAS is stronger than that between IMH and AD, as if they were “two sides of the same coin”.

### Informed consent statement

Written informed consent for the case to be published (incl. images, case history and data) was obtained from the patient for publication of this case report, including accompanying images.

## Learning points

1. Pain at the chest or back in an old female with cardiovascular risk factors and elevation of D-dimer should rise suspicion for AIH.
2. CT represents the gold standard in the diagnosis and follow-up of AIH and plays an important prognostic role.
3. Since the lack of univocal guidelines, patients with stable haemodynamics and no complications might benefit from medical therapy only, whereas patients with acute ULPs, wide intimal erosions, severe pain or hypertension, augmenting pleural effusion or high MAD should undergo prompt surgical/endovascular treatment.
4. Although associated with improved aorta-specific survival and delayed disease progression, TEVAR may complicate with high-mortality retrograde aortic dissection; for this reason, preemptive treatment should be performed only after a careful risk-benefit assessment.
